# Recent advances in understanding and managing gout

**DOI:** 10.12688/f1000research.9402.1

**Published:** 2017-03-10

**Authors:** Talia F. Igel, Svetlana Krasnokutsky, Michael H. Pillinger

**Affiliations:** 1 The Division of Rheumatology, Department of Medicine, New York University School of Medicine, New York, NY, USA; 2 The School of Clinical Sciences, Faculty of Medicine, Nursing and Health Sciences, Monash University, Melbourne, VIC, Australia

**Keywords:** gout, urate-lowering therapy, gout treatment

## Abstract

Gout is the most common crystal arthropathy and the leading cause of inflammatory arthritis. It is associated with functional impairment and, for many, a diminished health-related quality of life. Numerous studies have demonstrated the impact of gout and its associated conditions on patient morbidity and mortality. Unfortunately, gout remains under-diagnosed and under-treated in the general community. Despite major advances in treatment strategies, as many as 90% of patients with gout are poorly controlled or improperly managed and their hyperuricemia and recurrent flares continue. The introduction of novel urate-lowering therapies, new imaging modalities, and a deeper understanding of the pathogenesis of gout raise the possibility of better gout care and improved patient outcomes. Here, we spotlight recent advances in the diagnosis and management of gout and discuss novel therapeutics in gout treatment.

## Introduction

Gout incidence and prevalence have surged in recent years ^1^, reflecting population risk factors and the cultural transmission of predisposing habits of diet and behavior. Notwithstanding refined management guidelines, multiple effective medications, and improved physician understanding of treatment protocols, too many patients are still not meeting therapeutic goals ^2^. Fortunately, the rising prevalence of gout has brought a renewed interest in its biology, diagnosis, and treatment. Here, we review some recent advances in gout, including the introduction of novel therapeutics, the role of genetic screening, and the development of new gout classification and management guidelines.

## New biology: renal handling and the basis of hyperuricemia

Serum urate (sUA) levels are determined by the balance of metabolic production and excretion through the gastrointestinal tract and, most prominently, the kidneys. Among individuals who have primary hyperuricemia (that is, no acquired causes of urate overproduction or chronic kidney disease), upwards of 90% have urate elevation as a consequence of inadequate excretion ^3^. Recent genetic and physiologic studies have expanded our insight into the mechanisms through which uric acid is transported across the renal tubule ^4^. Although close to 100% of urate passing through a healthy kidney is filtrated by the glomerulus, only 5% to 10% is actually excreted ^5^. Among gout patients who are “primary underexcreters”, this number is even lower, ranging from 3% to 5% ^6^. The fractional excretion of urate (FEUA) tends to increase in response to rising sUA levels, providing a mechanism for sUA adjustment in response to serum loads. However, FEUA appears to be less responsive to sUA changes at higher sUA ranges and in the setting of primary under-excretion (that is, intrinsically low FEUA). In particular, the renal excretory system of patients with gout may be less responsive to rising sUA levels, reiteratively contributing to the pathogenesis of hyperuricemia ^7^.

Urate handling at the kidney occurs primarily in the proximal convoluted tubule (PCT), where transporters function either to reabsorb (for example, URAT1, OAT4, OAT10, and GLUT9) or secrete (for example, NPT1 and 4, MRP, and OAT1, 2, and 3) uric acid across the tubular endothelium. Among the reabsorbing transporters, URAT1 is central to maintaining sUA levels ^6^. Patients with deficiencies or inactivating mutations of the URAT1 transporter demonstrate markedly lower sUA levels compared with healthy controls ^8^, and drugs such as probenecid, losartan, and lesinurad (see “Lesinurad” section below) lower sUA and increase the fractional excretion of uric acid by inhibiting URAT1. Genome-wide association studies (GWAS) implicate genetic variants in URAT1, OAT4, OAT10, and GLUT9 in the development of hyperuricemia ^9– 12^, suggesting the possibility that patients with hyperuricemia have gain-of-function variants of these transporters that promote the retention of uric acid. Some drugs that cause hyperuricemia (for example, pyrazinamide) appear to function by promoting the retentive activity of the pumps, particularly URAT1. On the other hand, though it is less firmly established, GWAS suggest that variants in the secretory uric acid transporters NPT1 and 4, MRP, and OAT1–3 are also associated with hyperuricemia, presumably implying a loss-of-function state allowing sUA to accumulate.

The mechanisms of urate excretion from the intestine have been less well studied but may be of increased importance in patients whose renal excretion of uric acid is impaired. Recent GWAS data have implicated loss-of-function variants in a secretory pump, ABCG2, as a possible cause of hyperuricemia. Although ABCG2 was initially found to be expressed in the renal PCT, more recent studies suggest that it is much more highly expressed in the intestine, possibly providing insight into the mechanisms of gastrointestinal urate excretion ^6, 13^.

## New algorithms: diagnosis and classification

Historically, the diagnosis of gout focused on the acute arthritic state and did not consider the potential for chronicity. Proposed classification criteria demonstrated suboptimal sensitivity and specificity, were never validated, or did not incorporate advances in imaging modalities. In 2015, the American College of Rheumatology (ACR) and the European League Against Rheumatism (EULAR) jointly published validated classification criteria that encompass acute and chronic aspects of gout, recent imaging advances, and weighting to maximize sensitivity and specificity ^14^. These criteria permit improved enrollment of patients with gout into studies and provide a structure that can inform clinical diagnosis. Under the new algorithm, the documented presence of monosodium urate (MSU) crystals in a symptomatic joint or tophus is a sufficient criterion for classifying gout. If these criteria are not met, a scoring system is applied that reflects characteristics of acute and chronic gout, including recent advances in imaging (see Table 1 as well as the “New views: imaging” section below). A score of at least 8 indicates gout ^14^. A convenient web-based “gout classification calculator” based on these criteria has been released by the University of Auckland in New Zealand ( http://goutclassificationcalculator.auckland.ac.nz/).

**Table 1.** Scoring system for classification of gout.

Criteria	Category	Score
Pattern of joint/bursa involvement	Ankle or midfoot	1
	First metatarsophalangeal joint	2
Episodic symptoms • Erythema • Pain or tenderness • Functional disability	One symptom	1
	Two symptoms	2
	Three symptoms	3
Time course (at least two present): • Time to maximal pain is less than 24 hours • Resolution of symptoms in not more than 14 days • Complete resolution (to baseline) between episodes	One typical episode	1
	Recurrent typical episodes	2
Clinical evidence of tophus	Present	4
Serum urate	<4 mg/dL	−4
	6–8 mg/dL	2
	8–<10 mg/dL	3
	≥10 mg/dL	4
Synovial fluid analysis	MSU negative	−2
Imaging evidence of serum urate deposition in symptomatic joint or bursa: • Ultrasound: double-contour sign or • Dual-energy computed tomography: serum urate deposition	Present (either modality)	4
Imaging evidence of gout-related damage: • At least one erosion present in conventional radiography of hands or feet or both	Present	4

Adapted from the American College of Rheumatology/European League Against Rheumatism 2015 Gout Classification Criteria ^14^.

## New views: imaging

Recent advances in technology, together with a better understanding of the pathophysiology of gout, have led to better non-invasive tools facilitating the diagnosis and management of gout. The accelerating use of ultrasound and dual-energy computed tomography (DECT) is contributing to improvements in gout diagnosis, study, and management.

**Figure 1.**
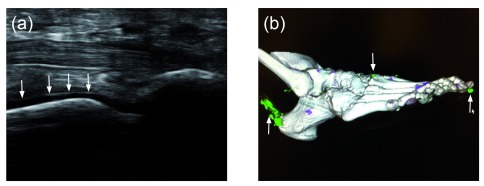
New imaging modalities for demonstrating serum urate deposition. ( **A**) Musculoskeletal ultrasound of a first metatarsal phalangeal joint (plantar longitudinal view) demonstrating a classic “double contour sign” (arrows), indicating the deposition of monosodium urate (MSU) crystals on the cartilage surface of the metatarsal head. ( **B**) Dual-energy computed tomography of a foot. Green areas indicate MSU deposition, and arrows indicate the presence of MSU deposition at the first distal interphalangeal joint, at the carpal metacarpal joint, and along the Achilles tendon.

The appropriate combination of symptoms on history—together with ultrasound findings of tophi, effusions with “snowstorm appearance”, and the pathognomonic “double contour” sign (deposition on the surface of articular cartilage)—may approach the sensitivity and specificity of joint aspiration for crystal examination and potentially preclude invasive intervention in at least some patients with gout ( Figure 1a) ^15– 17^. Although its use is currently limited by cost and availability, DECT can provide an accurate quantification of MSU crystal aggregates in both joints and soft tissues and permits the identification of deposits not appreciated by clinical examination. In the future, DECT may permit both recognition of occult deposits (total body burden) and monitoring of therapy to establish endpoints based on urate burden resolution ( Figure 1b) ^18^. The most up-to-date DECT technology (dual-source CT) results in radiation exposure that is no greater than that of conventional CT ^19^, with a single study of one extremity providing radiation exposure approximately equivalent to 4 months of natural background radiation. Nonetheless, this level of radiation exposure may limit the use of DECT on a recurring basis, as would be necessary when monitoring therapy. Regular CT, nuclear medicine, and magnetic resonance imaging have demonstrated utility in assisting the diagnosis of gout, especially in atypical presentations or cases managed by inexperienced providers.

Use of these imaging modalities has led to a shift in the paradigm about how gout begins. Formerly, most practitioners assumed that the first acute attack of gout was preceded by hyperuricemia and would be followed later by tissue MSU crystal deposition. However, both ultrasound and DECT have demonstrated the presence of MSU deposition in patients with hyperuricemia, even before the first gout attack ^18^. These observations suggest that by the time a patient has a first gout attack, he or she has already deposited MSU crystals in joints and tissues that will need to be depleted in the process of chronic gout management ^20^.

## New drugs

The field of gout therapeutics came to a virtual standstill in the latter half of the 20th century, during which no new drugs were approved for clinical use. In contrast, the early 21st century has witnessed a renaissance of gout therapy, beginning with the development of the xanthine oxidase inhibitor (XOI) febuxostat. It is a mark of the advancement of gout care in the last decade that, in this review, we will consider febuxostat to be an “old advance” and not discuss it further.

### New anti-inflammatory strategies

**Figure 2.**
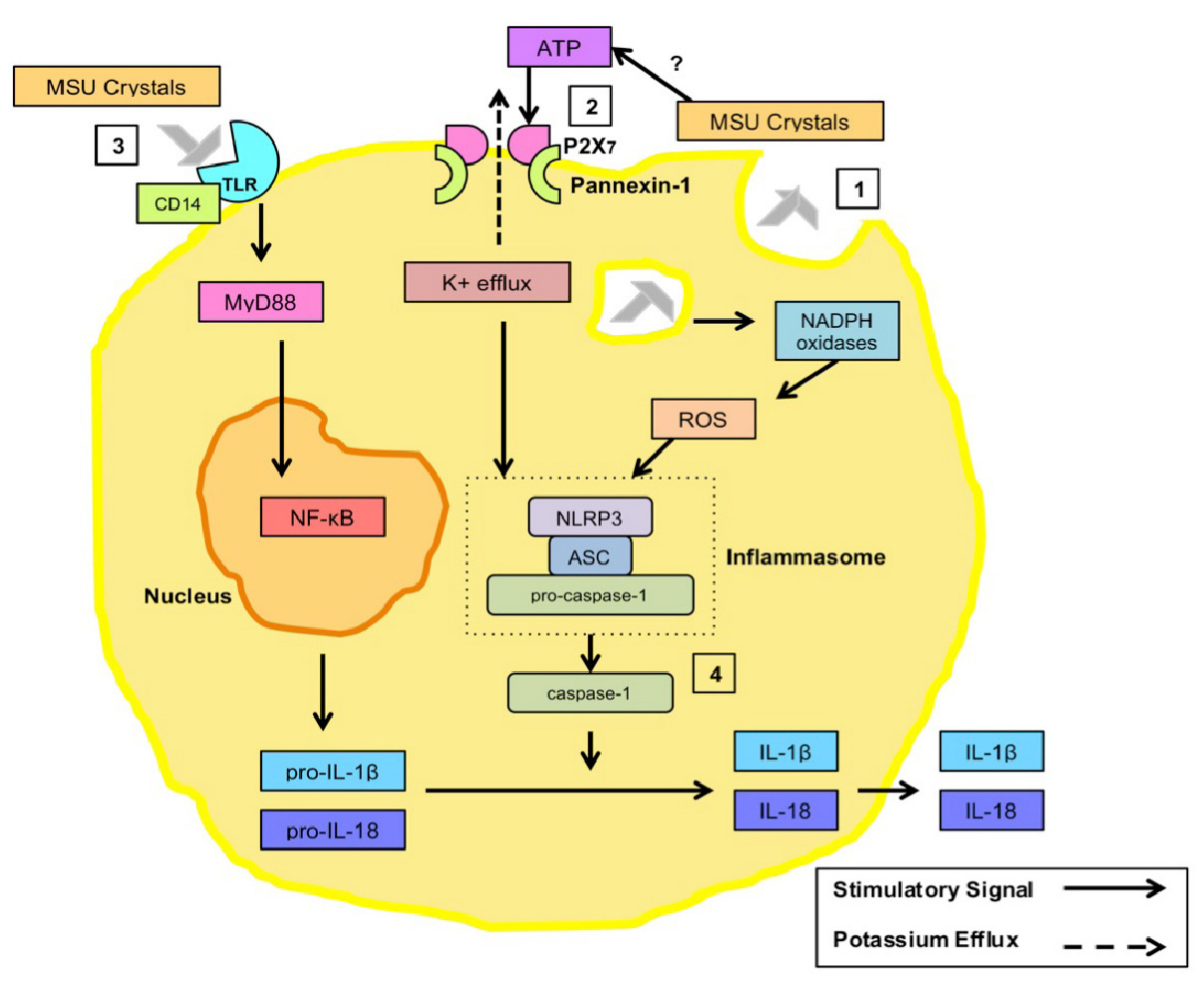
Activation of the NLRP3 inflammasome and the production IL-1β. ( **1**) Monosodium urate (MSU) crystal phagocytosis stimulates the NADPH (nicotinamide adenine dinucleotide phosphate) oxidase to generate reactive oxygen species that in turn can activate the NLRP3 (NOD-like receptor protein 3) inflammasome. ( **2**) MSU crystals may also stimulate the secretion of ATP, which can engage and activate the purinergic receptor P2X7, resulting in recruitment of pannexin-1 channels. The resultant rapid efflux of potassium, and the lowering of intracellular potassium, can also trigger inflammasome activation. ( **3**) Concurrently, MSU crystal interactions with Toll-like receptors (TLRs) on the cell surface stimulate the production of pro-IL-1β via MyD88- and NF-κB-dependent pro-IL-1β gene transcription. ( **4**) Once stimulated, the NLRP3 inflammasome’s enzymatic effector caspase-1 cleaves the pro-IL-1β to biologically active IL-1β. IL-1β is then secreted from the cell into the extra-cellular fluid of the site of inflammation. ASC, apoptosis-associated speck-like protein containing a caspase recruitment domain; IL-1β, interleukin-1 beta; NF-κB, nuclear factor-kappa B; NLRP3, NOD-like receptor protein 3; ROS, reactive oxygen species; TLR, Toll-like receptor.

Interleukin-1 beta (IL-1β) is synthesized on ribosomes as pro-IL-1β, an inactive molecule whose expression can be upregulated by multiple inflammatory stimuli. When converted to its active state by the NOD-like receptor protein 3 (NLRP3) inflammasome, IL-1β orchestrates much of the crystal-induced inflammatory response seen in acute gout ^21– 23^. The NLRP3 inflammasome is a multi-molecular complex composed of NLRP3, pro-caspase-1, and the adapter ASC (apoptosis-associated speck-like protein containing a caspase recruitment domain). Pathogen-derived or endogenous danger signals activate the NLRP3 inflammasome, leading to caspase-1 activation and activation and secretion of IL-1β ^24, 25^. The essential role of the NLRP3 inflammasome in acute gout attacks was recognized less than a decade ago, and the mechanisms through which MSU crystals activate the NLRP3 inflammasome are still under study ( Figure 2). Appreciation of the centrality of IL-1β to gouty inflammation has led to the off-label use of anti-IL-1β therapies for patients who do not adequately respond to or cannot tolerate traditional gout medications.

***Canakinumab.*** Canakinumab, a monoclonal antibody, neutralizes IL-1β to suppress inflammation. Canakinumab is US Food and Drug Administration (FDA)-approved for cryopyrin-associated periodic fever syndromes, Muckle-Wells syndrome, familial cold auto-inflammatory syndrome, and systemic idiopathic juvenile arthritis. Phase 3 trials of canakinumab by Schlesinger *et al*. demonstrated its efficacy in acute gout and for prophylaxis during sUA-lowering therapy (ULT) ^26, 27^. The FDA declined to approve canakinumab for acute gout therapy, citing concerns about the use of a long-acting immunosuppressant for an ostensibly short-term condition. In contrast, the European Medicines Agency approved canakinumab for the same indication.

***Anakinra.*** Anakinra is a recombinant human IL-1β receptor antagonist that is FDA-approved for rheumatoid arthritis and neonatal-onset multi-system inflammatory disease. To date, randomized controlled trials assessing anakinra’s efficacy in the management of gout are lacking ^28^, but case series and uncontrolled trials support its efficacy ^29, 30^. In practice, anakinra has been the preferred off-label anti-IL-1β strategy among experienced “goutologists”, based on its relatively short half-life and lower cost compared with canakinumab.

### New approaches to serum urate lowering

Because hyperuricemia is the underlying condition promoting gout, long-term treatment of gout almost always involves the therapeutic lowering of serum and tissue sUA levels. Several new ULTs are finding their way into the pharmacopaeia.

***Pegloticase.*** Pegloticase is a recombinant, pegylated uricase that degrades uric acid ^31^. Approved by the FDA in 2010, pegloticase is indicated for the treatment of hyperuricaemia in adults with chronic or tophaceous gout refractory to conventional ULT. Pegloticase is administered intravenously every 2 weeks. Studies confirm the ability of pegloticase to rapidly and dramatically lower sUA and to promote the often-dramatic resolution of tophi ^32^.

Several safety considerations arose during randomized controlled trials of pegloticase. As for all ULTs, pegloticase administration transiently raises the risk of gout flares. Therefore, gout flare prophylaxis is recommended for at least the first 6 months of pegloticase therapy. Pegloticase should be avoided in patients with glucose-6-phosphate dehydrogenase (G6PD) deficiency, as its action generates oxidants that may increase the risk of hemolysis and methemoglobinemia in such individuals. Because of infusion-associated volume loads, pegloticase should also be avoided in patients with uncompensated heart failure ^33^.

The biggest safety concern, and the most recurring cause of discontinuation in the trials, is the risk of infusion reactions. These reactions are generally mild but may be severe and necessitate pre-medication with glucocorticoids. Because both loss of drug efficacy and most infusion reactions reflect the development of anti-pegloticase antibodies (ironically, mainly directed at the polyethylene glycol portion, the very modification added to prevent such reactions ^34^), the risk of reactions can be greatly reduced by discontinuing pegloticase in patients whose sUA exceeds 6.0 mg/dL prior to infusion on two consecutive occasions ^35^. Oral ULTs are contraindicated during pegloticase use, to prevent masking any loss of pegloticase efficacy ^35^. Though highly effective, such a safety strategy does not solve the overall treatment problem for patients with severe gout who need but cannot tolerate the agent; therefore, investigations to try to identify strategies to reduce the risk of pegloticase intolerance are ongoing ^36^.

***Lesinurad.*** Lesinurad is a selective, highly potent uric acid reabsorption inhibitor. Lesinurad reduces sUA by inhibiting both the sUA-anion exchanger transporter 1 (URAT1) and the organic anion transporter 4 (OAT4), which are involved in the reabsorption of sUA across the renal proximal tubule ^37^. In contrast to the older uricosuric probenecid, lesinurad is more potent and remains effective even in moderate renal insufficiency. In 2015, lesinurad gained FDA approval as a second-line treatment for gout patients who have failed to meet target sUA despite treatment with a traditional XOI ULT (that is, allopurinol or febuxostat).

The Combination Study of Lesinurad in Allopurinol Standard of Care Inadequate Responders (CLEAR 1 and CLEAR 2) assessed the efficacy of lesinurad (200 or 400 mg daily) as a ULT. The addition of lesinurad to standard allopurinol care increased the proportion of patients successfully meeting sUA targets by as much as 2.5-fold ^38^. Similarly, the CRYSTAL study compared combination therapy with lesinurad and febuxostat with febuxostat monotherapy in the treatment of hyperuricemia and resolution of tophi. Combination therapy increased the number of patients achieving target sUA below 5 mg/dL compared with febuxostat alone and resulted in improved tophus resolution ^39^. In both studies, the 400 mg dose of lesinurad was associated with an increased frequency of serum creatinine elevations compared with the 200 mg dose or with XOI alone. Another study, the LIGHT (lesinurad monotherapy in gout patients intolerant to XOIs) study, also demonstrated a potential for creatinine increases when using the 400 mg dose as monotherapy ^40^. For this reason, lesinurad is approved only at the 200 mg dose and only in conjunction with an XOI. Baseline assessment and periodic testing of renal function are required, particularly for patients with creatinine clearance below 60 mL/min.

***Arhalofenate.*** Arhalofenate is a pipeline drug with a dual mechanism of action. Patients initiating ULT are routinely prescribed concurrent anti-inflammatory prophylaxis to reduce the risk of gout attacks precipitated by the sUA-lowering process itself. Historically, all gout medications have been either anti-inflammatory or sUA-lowering. In contrast, arhalofenate, a peroxisome proliferator-activated receptor-gamma (PPAR-γ) partial agonist, demonstrates dual ULT and anti-inflammatory effects. Specifically, arhalofenate inhibits expression of IL-1β while inhibiting renal reabsorption of uric acid at the URAT1, OAT4, and OAT10 transporters ^41^.

A randomized controlled trial assessed the effectiveness of arhalofenate compared to allopurinol and placebo. Though demonstrating a greater capacity for sUA lowering than placebo, it did not show superiority over allopurinol. Similarly, arhalofenate did not appear to be as effective as traditionally used anti-inflammatories ^42^. Nonetheless, the possibility that the dual action of arhalofenate permits single-drug regimens for at least some patients with gout could improve compliance for this disease in which patients—and their physicians—have been notoriously non-compliant with treatment.

## New treatment guidelines

In 2012, the ACR published its first gout treatment guidelines. These guidelines reasserted that chronic gout needs chronic treatment and integrated the issues of anti-inflammation, ULT, and lifestyle risk management. With an emphasis on early management of gout, the guidelines promote evidence-based best practice, improve quality of therapy, and enhance patient safety.

Recommendations were reached after a formal review procedure by a multi-center, international team of gout physician experts. Innovations include guidance on the proper time to initiate ULT (in the setting of two attacks within the same year or after one attack in patients with stage 2 or greater renal disease, tophi, or kidney stones), an emphasis on treat-to-target strategies (an initial target of less than 6.0 mg/dL and lower as needed to control attacks or resolve tophi or both), the use of uricosuric agents and pegloticase as second- and third-line ULTs, and a sidelong endorsement of off-label anti-IL-1 biologics when conventional anti-inflammatory strategies fail ^28, 43^.

With the introduction of novel gout medications and new information regarding traditional gout therapy, the EULAR has recently published its own gout management guidelines. In contrast to the ACR, the EULAR recommends consideration of ULT initiation in every patient with a definite diagnosis of gout at first presentation. This recommendation advances the initiation of ULT to an earlier point in the disease, reflecting the awareness that by the time a patient’s first attack occurs he or she should already be considered to have a chronic disease, potentially with occult MSU crystal deposition, and that clearance of MSU crystals may be more difficult once a larger crystal burden has become established ^44^. Indeed, recent imaging studies suggest that MSU deposition can be identified in a significant percentage of patients with hyperuricemia, even before their first gout attack ^45^.

While the evidence base regarding early ULT intervention continues to evolve, recent studies suggest that delayed initiation of ULT may increase the cardiovascular and renal risk associated with greater exposure to hyperuricemic states (see New Risks—and Benefits?—of Hyperuricemia, below) ^46, 47^ and that prompt and comprehensive ULT may reduce the risk of associated cardiovascular and renal morbidity ^48^. However, current studies are either retrospective or too small to be considered pivotal, and the notion of early ULT intervention remains somewhat controversial, particularly outside of the rheumatology community. The EULAR recommendation to consider initiating ULT even after a single gout attack is based primarily on expert opinion, and controversy recently erupted with the release of American College of Physicians gout treatment guidelines, which fail to enthusiastically recommend chronic ULT use or treat-to-serum urate target strategies in patients with chronic gout ^49^.

## New genetics for screening

The human leukocyte antigen B (HLA-B), a cell surface protein involved in the recognition and presentation of foreign antigens, is critical to immune defense. One variant of this gene, the HLA-B*58:01 allele, has been strongly linked to increased (>100-fold) risk for severe cutaneous and systemic adverse reactions upon treatment with allopurinol. HLA-B*58:01 has been most commonly associated with Asian cohorts. Among Han Chinese ^50^ and Thai ^51^ individuals, the allele has been found in 100% of patients with allopurinol hypersensitivity reactions. In Korean ^52^ patients, the allele was also present in 80% of allopurinol hypersensitivity reactions, a number far greater than the 12% seen in healthy controls.

Therefore, the current ACR gout management guidelines recommend testing all patients of Han Chinese and Thai ancestry and all patients of Korean descent with at least stage 3 renal failure ^43^. The Clinical Pharmacogenomics Implementation Consortium recommends that allopurinol not be prescribed to patients positive for HLA-B*58:01 ^53^. Pharmacoeconomic analyses of HLA-B*58:01 genotyping in high-risk patients suggest that such a testing strategy is cost-effective ^54^.

Very recent studies by Lu *et al*. and others have addressed the fact that, like some Asian populations, African-Americans may have a higher prevalence of HLA-B*58:01. In a recent multi-center study, the risks of allopurinol hypersensitivity reactions in the Asian and African-American subpopulations were 12 and 5 times greater, respectively, compared with Caucasians. These figures were concordant with the observed incidence ratios of the HLA-B*58:01 allele in the particular patient populations (7.4% in Asian cohorts, 4% in African-American cohorts, and 1% in Caucasian cohorts) ^55^. The large number of African-Americans potentially genetically predisposed to allopurinol hypersensitivity associated with the HLA-B*58:01 gene raises the question of whether widespread testing of this population is warranted.

Recent data have alluded to a potential risk reduction in allopurinol hypersensitivity syndrome with graded dose introduction of ULT ^56^. As a result, ACR treatment guidelines recommend starting all patients on allopurinol at a low dose and titrating up gradually until a target sUA is achieved ^43^. Whether the degree of risk reduction obtained using this strategy is sufficient to allow practitioners to eschew universal HLA-B*5801 testing in populations at high risk for allopurinol hypersensitivity has not been directly studied.

## New risks—and benefits?—of hyperuricemia

There is growing recognition within the gout community of a potentially causative relationship between hyperuricemia and cardiovascular disease, and multiple large population studies have examined this association. Despite differences in study populations and varying conclusions, almost all of the studies confirmed that hyperuricemia is an independent risk factor for adverse cardiovascular outcomes ^57^. A similar potential adverse effect of hyperuricemia has been examined rigorously in connection with chronic kidney disease ^58^. Studies suggest that high sUA concentrations impact the level of kidney damage and also are associated with a larger risk of secondary hypertension ^58^. A number of small interventional trials, and large population-based studies in patients with either hyperuricemia or gout, suggest that treatment with ULTs may reduce the risk of these adverse outcomes ^59– 61^. Pivotal clinical trials will be needed to determine whether ULT treatment is warranted to lower co-morbid risk in hyperuricemic individuals either with or without gout.

Whereas rheumatologists and their patients view high sUA levels and the acute attacks they bring as inherently undesirable, some neurologists believe that the anti-oxidant properties of sUA may have neuroprotective benefits against Alzheimer’s and possibly other neurodegenerative diseases. Within the bloodstream—and presumably within the central nervous system—sUA provides the greatest extra-cellular contribution to anti-oxidation.

Using data obtained from the Health Improvement Network, an electronic medical record database representative of the population of the United Kingdom, Lu *et al*. studied the relationship between gout/hyperuricemia and Alzheimer’s disease. In their age-matched, sex-matched, body mass index-matched, and entry time-matched cohort study, gout was inversely associated with the risk of developing Alzheimer’s disease. Univariate and multi-variate hazard ratios for Alzheimer’s disease diagnosed in patients with gout were 0.71 (95% confidence interval [CI] 0.62 to 0.80) and 0.76 (95% CI 0.66 to 0.87), respectively ^62^. Similarly, sUA levels in patients with multiple sclerosis are significantly lower than those of healthy controls, and few if any cases of co-morbid gout and multiple sclerosis have been reported ^63, 64^. Other studies have tentatively identified similar relationships between gout and sUA and both Parkinson’s ^65^ and Huntington’s ^66^ diseases. However, not all studies have recognized this possible beneficial effect ^67^.

Since the putative neuroprotective relationship between uric acid and central nervous system disorders is a relatively new concept, there is currently no guidance as to what specific level of sUA, if any, might represent an appropriate balance between neuroprotective benefit and gout/gout co-morbidity risk. Therefore, further studies are required to confirm that higher sUA levels actually provide a neurologic benefit and to re-evaluate what constitutes a “healthy” sUA level in both gout and non-gout patients. Importantly, there are currently no data to suggest that urate lowering in patients with hyperuricemia increases the likelihood of either developing a neurodegenerative disease or worsening such a process if already present.

## Conclusions

With recent advances in our understanding of the pathophysiology of hyperuricemia and crystal inflammation, gout has once again come to the fore as a disease bearing serious implications and requiring intricate therapy. New appreciation of the centrality of IL-1β and the inflammasome and greater insight into the transporters crucial to renal sUA handling have paved the way for the introduction of novel gout therapeutics with diverse modes of action. Similarly, with growing understanding of the genetics behind gout and the multiple functions of sUA, recommendations for the management of gout are undergoing evolution and refinement.

With improvements in both patient and provider comprehension of gout as both an acutely debilitating and chronic disease, developments in gout pharmaceutics, and ongoing research into the disease biology, the rheumatologic community continues to make significant headway in producing adequate control of sUA and prevention of acute gout flares.
